# Gender and Psychological Well-Being

**DOI:** 10.3390/ijerph16193531

**Published:** 2019-09-20

**Authors:** M. Pilar Matud, Marisela López-Curbelo, Demelza Fortes

**Affiliations:** Department of Clinical Psychology, Psychobiology and Methodology, Universidad de La Laguna, 38207 La Laguna, Spain; marlocur@ull.edu.es (M.L.-C.); demel81@hotmail.com (D.F.)

**Keywords:** psychological well-being, gender, masculinity, femininity, occupation

## Abstract

*Background*: Research has consistently reported gender differences in mental health, but studies on differences in psychological well-being between women and men have not yielded conclusive results. The aim of this study was to examine the relevance of gender to the psychological well-being of adult individuals. A cross-sectional study with a sample of 1700 men and 1700 women from the general Spanish population was conducted. Their ages ranged from 21 to 64 years, and they were assessed with Ryff’s Psychological Well-Being Scales and the Bem Sex Role Inventory. *Results*: Men scored higher than women in self-acceptance and autonomy, and women scored higher than men in personal growth and positive relations with others. The most relevant variable in the psychological well-being of both women and men was high masculinity. Other relevant variables in women’s well-being were high femininity, not having a manual occupation, not being homemakers, and professional occupation. Men´s well-being also was higher in professional men and in men with a skilled non-manual occupation, men with high femininity and men who were not single, divorced or widowed. *Conclusions*: Adherence to traditional gender roles is relevant to the psychological well-being of women and men, and women and men whose self-concept includes both masculine-instrumental and feminine-expressive characteristics have greater well-being.

## 1. Introduction

Gender is an important social determinant of health [1] and gender-based analysis is necessary to improve women’s and men’s health and health care. Some health indicators have disclosed differences between women and men, so contends research conducted in many countries. Worldwide, female life expectancy is greater than male life expectancy [2]. Likewise, mental health reveals consistent differences between females and males [3,4]. Usually, it is found that women have, more frequently than men, internalizing disorders, such as depression [3,4,5] and psychological distress [6,7,8], while men have, more frequently than women, externalizing disorders, with higher antisocial and substance use disorders [3,4]. Also, worldwide, men’s suicide rates are higher than those of women [2]. 

Studies on gender differences in health have regarded life expectancy and the existence of diseases and health-related issues as indices of health status, based on the medical health model centered only on pathology and illness [9]. As stated by Seligman and Csikszentmihalyi [10], focusing on pathology, a perspective that has also prevailed in a major part of psychology, has generated a human-being model lacking the positive features which make life worthwhile. This has changed in the last few decades, and the relevance of psychological well-being has been asserted [9,11]. Psychological well-being refers to living life in a complete and satisfying way and to the development and self-realization of the individual [12,13]. Within this perspective, Ryff’s model and measure of psychological well-being [13] is the most widely used instrument. Ryff’s model includes six dimensions: (1) Self-acceptance, which refers to having a positive attitude toward oneself and past experiences, recognizing and accepting one’s own characteristics; (2) positive relations with others, which refers to having true, warm, and satisfactory relations with other people and being concerned for their well-being; (3) autonomy, which basically refers to the extent to which the individual is self-determining and independent; (4) environmental mastery, which refers to the individual’s capacity to handle and control the environment, taking advantage of the opportunities the environment offers to fulfil one’s needs and values; (5) purpose in life, which includes having goals and objectives in life, and feeling that life has sense; and 6) personal growth, which refers to the feeling of continuous evolution and growth, and having the feeling that one is developing one’s own potential [13,14]. After reviewing the research that drew on this model of well-being for more than two decades, Ryff [15,16] stated that psychological well-being was not only a matter of psychological flourishing and self-realization, but also important in physical health. There exists evidence that it plays a protective role against a number of illnesses and disabilities through the optimal regulation of various physiological and neurological systems. Longitudinal research has proved that persistently high well-being was associated with individuals whose subjective health was better, and who reported less chronic conditions, fewer symptoms and lower levels of functional disability than in the case of individuals with low levels of well-being [17]. In addition, poor psychological well-being is significantly and positively correlated with mortality [18,19]. 

Currently, it is recognized that well-being, in addition to being multidimensional, has a dynamic nature, and is influenced by personal and cultural factors [20], and the probability of high well-being increases as education and job status increases [21,22,23,24]. It has been suggested that cultural variations in well-being are associated mainly with the different conceptions of the self and the relations that exist in the different cultures [25,26]. Collectivistic cultures feature an interdependent construal of the self, and place emphasis on attention and adjustment to others, and harmonious interdependence with them; these are factors that they prioritize over their personal needs. On the contrary, individuals in individualistic cultures try to maintain their independence of others, and discover and express their unique attributes; hence, they frequently prioritize their personal goals over the goals of their group [27]. Even though it has been proposed that the type of interdependence can be different, based more on individual and close relations than on group membership [28], the conception of the self as independent or interdependent is central to gender socialization.

Although research has reported that women and men are similar in most psychological traits [29,30], the majority of societies consider they are essentially different and should occupy different roles. Customary theories approaching differences in gender roles posited that those differences were normal and healthy as they mirrored accepted standards of social propriety to each gender. For instance, traditional formulations “suggested that adoption of the sex roles appropriate to one’s male or female gender is developmentally desirable” ([31], p. 777). However, after more than three decades of research, the benefits versus costs of being gender typed still remain unclear [32]. It seems rigorous compliance with masculine and feminine roles may narrow the range of potential behaviors and choices of women and men, which would in itself limit the development of those personal characteristics that do not conform to social expectations about gender propriety [33,34]. Moreover, although there are major methodological problems and different conceptualizations and measures of masculinity [35,36], masculinity has been found to be more associated with the well-being of men and women than femininity [32,37].

Studies about the differences between women and men on well-being have not yielded consistent outcomes [38]. Results have demonstrated few gender differences in psychological well-being, although women reported having experienced positive and negative emotions with greater frequency and intensity than men [39]. Although literature has shown differences between women and men in some psychological well-being dimensions [26,40,41,42], such differences generally vary depending on other factors such as age, culture, or roles played [21,26]. One difference between women and men that has been consistently found is the higher score of women in positive relations with others [14,21,26]. It has also been found in different cultures that women scored lower than men in self-acceptance and autonomy [21,26], though in the Karasawa et al. study [26], the differences between women and men in autonomy only appeared in the early decades of adulthood. The overall aim of this study was to analyze the relevance of gender to the psychological well-being of adult individuals. The specific objectives of the work were the following: (1) To know whether there are differences between women and men in psychological well-being; (2) to know the association between masculinity and femininity with the psychological well-being of women and men; and (3) to study the relevance of the occupation to the psychological well-being of women and men. Based on the reviewed literature, we formulated the following hypotheses:

(1) Women will score higher than men in positive relations with others.
(2) Men will score higher than women in self-acceptance and autonomy.
(3) Women and men with a higher job level will have more psychological well-being than less qualified workers.
(4) Masculinity will be more associated with the psychological well-being of men and women than femininity.

## 2. Materials and Methods 

### 2.1. Participants and Procedure

The sample consisted of 3400 individuals from the general population, specifically 1700 women and 1700 men aged between 21 and 64 years [age mean (*M*) = 41.87, standard deviation (*SD*) = 11.29] with similar age and educational levels for both women and men. All the individuals who participated in the study were volunteers, and did not receive any monetary compensation for their participation. Access to the sample was through employment centers and community associations located in all Spanish autonomous communities, as well as through social networks (generally known persons, friends, neighbors and relatives) of psychology and sociology university students trained in administering those tests, who received course credits for that task. The sample of the present study was extracted from a larger one on the basis that they comply with the following conditions: (1) Aged between 21 and 64 years; (2) similar age and educational level for women and men; and (3) no missing values in the database for gender (woman or man), the Ryff’s Psychological Well-being Scale or the Bem Sex Role Inventory (BSRI) Scale. After informed consent was obtained, the psychological inventories and a sociodemographic data collection sheet were self-completed through individual and manual responses in a booklet where these tests were printed. The researcher, or the research collaborator in charge of passing the test to that person, handed him/her the booklet and arranged with him/her a date and place for its collection once completed, trying to ensure that the time interval for completion did not exceed two weeks. We complied with American Psychological Association ethical standards in the treatment of the sample and no names or any other data identifying the participant were used in the tests. This research forms part of a larger study on gender and health and was positively evaluated by the Ethics of Research and Animal Well-Being Committee of the University of La Laguna (study approval number 2015–0170).

Table 1 displays the demographic characteristics of the women´s and men´s groups. Although there were no statistically significant gender differences in age or education, there were some differences in occupation, marital status and number of children.

### 2.2. Measures

Ryff’s Psychological Well-Being Scale in the version shortened by van Dierendonck [43]. We used the translated and validated version in Spanish, in which the authors found a structure consisting of six factors, with one second-order well-being factor [44]. This version consists of 38 items, with a response format ranging from 1 (totally disagree) to 6 (totally agree), which are structured in the following subscales: Self-acceptance, made up of 6 items with a Cronbach’s alpha of 0.79; positive relations with others, consisting of 6 items, with a Cronbach’s alpha of 0.78; autonomy, made up of 8 items, with a Cronbach’s alpha of 0.71; environmental mastery, consisting of 6 items, with a Cronbach’s alpha of 0.68; purpose in life, made up of 6 items, with a Cronbach’s alpha of 0.82; and personal growth, consisting of 6 items, with a Cronbach’s alpha of 0.71 [44]. In the sample of this study, Cronbach’s alpha coefficients were quite similar with coefficients, respectively, of 0.81, 0.80, 0.71, 0.64, 0.83, 0.69. The Cronbach’s alpha of all items on the scale, which constitute the second-order well-being factor, was 0.93.

Bem Sex Role Inventory (BSRI) [45]. The BSRI is one of the most commonly used instruments worldwide to assess the self-attribution of the personality traits deemed typical of each gender [6,46]. It comprises 60 items—adjectives or short sentences; from these, 20 refer to features so far regarded as masculine, which makes up the masculinity scale; another 20 relate to characteristics traditionally associated with femininity, thus forming the femininity scale; while the other 20 items constitute characteristics that can be attributed to both genders. The response format is a 7-point Likert scale ranging from 1 (never or almost never true) to 7 (always or almost always true). In the sample of this study, the Cronbach’s alpha of the 20 items on the masculinity scale was 0.83, and the Cronbach’s alpha of the 20 items on the femininity scale was 0.80.

In addition, a sociodemographic data collection sheet was used in which job information was also collected and the person was asked to indicate whether he/she was woman or man. If a person marked both options (woman and man) or did not indicate any, that subject was removed from the study.

### 2.3. Statistical Analysis

Cronbach’s alpha coefficient was used to assess the reliability of the psychological well-being and masculinity and femininity factors. To examine whether the women and men groups differed in terms of sociodemographic variables, Chi-square tests and *t* tests were conducted. The analysis of gender differences on the well-being scales was conducted by means of multivariate analyses of variance (MANOVA) and univariate analyses of variance (ANOVA). Effect size was calculated using Cohen’s *d* [47]. Pearson correlation analysis was conducted to examine correlations among masculinity and femininity with well-being factors. Hierarchical multiple regression was used to determine the relevance of masculinity and femininity and the occupation in women’s and men’s well-being. Correlations and regression analyses were separately performed for women and men. IBM SPSS Statistics for Windows version 22.0 (IBM Corp., Armonk, NY, USA) was used for all analyses.

## 3. Results

In the MANOVA, where the factor was gender (men, women) and the dependent variables were the six well-being scales, statistically significant differences were found, *F*(6, 3393) = 9.61, *p* < 0.001. The results of ANOVAs showed statistically significant differences in four of the well-being dimensions (see Table 2). Though the effect size was small, women had mean scores higher than men in positive relations with others and personal growth, while men scored higher than women in self-acceptance and autonomy.

Table 3 shows bivariate correlations, in the sample of women and men, of well-being scales with scores in masculinity and femininity. In both genders, masculinity was associated with greater psychological well-being. Femininity was also associated in both genders with higher scores in all well-being dimensions, except with autonomy. In both genders, the magnitude of the correlation coefficients was lower than those for masculinity in self-acceptance, environmental mastery, and purpose in life, as well as in personal growth for the women sample.

Two hierarchical regressions (one for each gender) were conducted to determine the relevance of masculinity and femininity and the occupation in the women’s and men’s psychological well-being. Age, number of children, and marital status as a dummy variable were entered in step 1 to control their effect. At step 2, masculinity and femininity were entered. Finally, at step 3, occupation as a dummy variable was entered. All variables, except dummy variables, were centered for lessening multicollinearity [48]. Table 4 displays the results for the women’s group and Table 5 for the men’s group. *R*s for regression were, in both groups, significantly different from zero in each model. The most relevant variables in the psychological well-being of the women’s group were high masculinity and, to a lesser extent, high femininity, not having a manual occupation, not being homemakers, a younger age, and a professional occupation (see Table 4). The most relevant variable in the psychological well-being of the men’s group was also high masculinity. Other relevant variables in men’s psychological well-being were a skilled, non-manual and professional occupation, high femininity, and not being single or divorced or widowed (see Table 5).

## 4. Discussion

The purpose of this study was to analyze the relevance of gender to the psychological well-being of adult individuals. Women and men groups had similar age and educational levels since chronological age and educational status define the individuals’ position in the social structure and both variables have been associated with well-being [15,49]. Although the effect size of differences was small, statistically significant differences were found between women and men in some psychological well-being dimensions, with men scoring higher than women in self-acceptance and autonomy, and with women scoring higher than men in personal growth and positive relations with others. Therefore, the first and second hypotheses of the study were confirmed. The existence of lower scores for women, as compared to men, in self-acceptance and autonomy, has also been found in studies conducted in individualistic countries, such as the United States [21,26] and in collectivistic countries such as Japan [26]. However, in this study, gender differences only occurred in the two early decades of adulthood researched (35−44 and 44−54), but not at an older age (55–64 years, and 65–74 years). Moreover, in the studies conducted by Ryff and colleagues, both in the United States and Japan [14,21,26], it was found that women score higher than men in positive relations with others. Women scored higher than men in personal growth in this study; these differences, though found in the first studies carried out by Ryff and colleagues [14], were not detected in subsequent research [21,26]. Even though we do not know the reason for the differences between the results of this study and those of the aforementioned studies, and the causes for which, in Spain, women score higher than men in personal growth, it may be the consequence of both the major social changes which have taken place in Spain in the last few years, and the differences in the social context between the countries. Spain occupies an intermediate place in the individualism versus collectivism dimension [25], and the aforesaid studies were conducted in the United States and Japan, two countries that score at the extremes of the dimension. Besides, in the last few decades, there have been major political and social changes in Spain, especially for women, the most remarkable of which has been access to education, an area where gender equality is detected, and access to employment, although labor force participation for women and men is unequal [50]. Although statistically significant differences have been found in four of the six dimensions of psychological well-being in this work, the effect size of such differences was very small. This may be due to the fact that the women and men in the study had similar educational levels. In studies performed in other countries, education and employment have proved to be relevant to well-being [23,41,51,52].

The third hypothesis predicted that women and men with a higher job level would have greater well-being than less qualified workers. This hypothesis was confirmed. In studies conducted in other countries, it has also been found that well-being is associated with employment and job level [23,52]. While in the study by Trzcinski and Holst [51] it was found that the effect of job status on well-being was stronger in men than in women, we have found, in this study, that job status is relevant to the well-being of both women and men. In fact, Spanish women who are homemakers or have a skilled or unskilled manual occupation present lower well-being than those with a higher job level.

The fourth hypothesis, which predicted that masculinity would be more associated with the psychological well-being of men and women than femininity was supported as well. In both genders, masculinity was positively associated with all the well-being dimensions. Even though femininity was also positively associated with all the well-being dimensions (except for autonomy), the proportion of variance in common with the well-being dimensions was lower than that for masculinity, except for positive relations with others. Regression analysis also showed that masculinity was more associated with psychological well-being than femininity. It has also been found in other studies that, although both dimensions are associated with well-being, the effect is higher for masculinity than for femininity [37,53]. When interpreting these associations, it is necessary to take into account that masculinity and femininity have been assessed by the BSRI [45]. The BSRI masculinity scale contains socially desirable characteristics that have been stereotypically associated with men, such as independence, assertiveness, strength, individualism or ambition. The femininity scale comprises characteristics stereotypically associated with women, such as empathy, tenderness, warmth or the need for affiliation. The results of the present study show that, although both types of characteristics are important for the well-being of women and men, those that imply independence, assertiveness, strength, individualism or ambition entail greater well-being for both men and women.

The results of this study have practical implications and they may be effective for the design of policies and programs aiming to enhance women’s and men’s wellbeing and to achieve greater gender equality. They help deconstruct gender stereotypes as the results suggest that the internalization of attributes and behaviors traditionally considered "masculine" are associated with greater psychological well-being in both women and men. The same applies to the attributes considered stereotypically “feminine”, although their association with the well-being of both women and men is smaller. Moreover, in opposition to gender stereotypes which have perpetuated that a woman’s highest priority concerns her family role whereas a man’s highest priority is occupation, this study has found that occupation is associated with well-being in both women and men. In addition, although men are stereotyped as self-oriented and women as other-oriented [54], the results of this study indicate that marital status is associated with well-being in the case of men but not in the case of women; in fact, men who had never married or divorced or widowed presented lower psychological well-being.

There are several limitations to the current study. First, when interpreting the associations found between masculinity and femininity and well-being, it is necessary to take into account that masculinity and femininity were assessed by the BSRI [45]. It has been proposed that such an instrument is limited to the assessment of the gender-related traits of agency and communion rather than complete measures of masculinity and femininity [55]. However, agency and communion are the core of gender stereotypes and these traits are central to the cultural framing of gender [56,57]. Other limitations of our study should also be taken into account. The sample, though large, has not been obtained through random sampling. All the participants lived in Spain, which can restrict the generalization of results to other countries. Further, it is a cross-sectional analysis and as such it can only show an association between variables, but not cause-and-effect relationships. Future research is needed to investigate the causal link between the variables addressed in this paper.

## 5. Conclusions

The data from this study supports the perspective that affirms an investment in gender ideals implies a limitation to the development of the whole potential of human beings [33,47]. Adherence to traditional gender roles and occupation are relevant to the well-being of Spanish women and men, and women and men whose self-concept includes both masculine-instrumental and feminine-expressive characteristics have greater psychological well-being. The results of this study have practical implications and they may be useful for the design of policies and strategies intended to increase the well-being of population and to achieve greater gender equality.

## Figures and Tables

**Table 1:** Demographic characteristics of the men and women groups.

	Men (*n* = 1700)		Women (*n* = 1700)		
Characteristic	*n*	%	*n*	%	*χ*^2^-Value
Education:					
Primary	569	33.80	565	33.40	
Secondary	522	31.10	493	29.20	
University	590	35.10	633	37.40	
No data	19		9		
					2.32
Occupation:					
Skilled/unskilled manual	648	38.10	517	30.40	
Skilled non-manual	647	38.10	502	29.50	
Professional	405	23.80	450	26.50	
Homemaker	0	0.00	231	13.60	
					266.40 ***
Marital status:					
Never married	547	32.20	454	26.70	
Married/cohabiting	1061	62.40	1032	60.70	
Divorced/widowed	92	5.40	214	12.60	
					57.68 ***
	*M*	*SD*	*M*	*SD*	***t*-Value**
Age	41.83	11.35	41.91	11.22	−1.90
Number of children	1.15	1.12	1.31	1.15	−4.19 ***

Note: *** *p* < 0.001.

**Table 2:** Means (*M*), standard deviations (*SD*) and comparisons for men and women for the well-being scales.

	Men (*n* = 1700)		Women (*n* = 1700)		*F*_(1, 3398)_	*d*-value
	M	SD	M	SD		
Self-acceptance	27.12	4.81	26.53	5.19	11.82 **	0.12
Positive relations with others	26.87	5.59	27.28	5.86	4.35 *	−0.07
Autonomy	35.86	5.81	35.38	6.02	5.6 2*	0.08
Environmental mastery	27.50	4.14	27.38	4.35	0.65	0.03
Purpose in life	28.15	4.87	27.86	5.19	2.73	0.06
Personal growth	27.83	4.33	28.25	4.36	7.85 **	−0.10

Note: * *p* < 0.05; ** *p* < 0.01. *d*-value = Cohens’s *d*

**Table 3:** Bivariate correlations between masculinity and femininity with well-being scales for the groups of women and men.

	Women		Men	
	Masculinity	Femininity	Masculinity	Femininity
Self-acceptance	0.31 ***	0.14 ***	0.30 ***	0.19 ***
Positive relations with others	0.19 ***	0.17 ***	0.15 ***	0.13 ***
Autonomy	0.36 ***	0.02	0.28 ***	−0.02
Environmental mastery	0.30 ***	0.14 ***	0.33 ***	0.19 ***
Purpose in life	0.33 ***	0.19 ***	0.38 ***	0.26 ***
Personal growth	0.30 ***	0.21 ***	0.31 ***	0.29 ***

Note: *** *p* < 0.001.

**Table 4:** Summary of the hierarchical regression with the psychological well-being factor as the dependent measure for the women’s group.

	Model 1		Model 2		Model 3	
	*β*	*t*-value	β	*t*-value	β	*t*-value
Age	−0.13	−3.86 ***	−0.11	−3.78 ***	−0.10	−3.45 **
Number of children	−0.01	−0.14	−0.01	−0.03	0.03	0.86
Never married	−0.01	−0.43	−0.02	−0.77	−0.02	−0.68
Divorced/widowed	0.01	0.20	−0.03	−1.49	−0.03	−1.48
Masculinity			0.36	15.76 ***	0.35	15.25 ***
Femininity			0.10	4.59 ***	0.11	4.86 ***
Skilled/unskilled manual occupation					−0.11	−4.29 **
Professional					0.07	2.58 *
Homemaker					−0.09	−3.31 **
Adjusted *R*^2^	0.01		0.17		0.19	
*R*^2^ Change	0.01		0.16		0.03	
ANOVA (*F*-value, df)	6.46 (4,1683) ***		57.86 (61,681) ***		46.02(91,678) ***	

Note: β = Standardized regression coefficient. *R*^2^ = percentage of explained variance. * *p* < 0.05; ** *p* < 0.01; *** *p* < 0.001. *t*-value = Student *t*-value

**Table 5:** Summary of the hierarchical regression with the psychological well-being factor as the dependent measure for the men’s group.

	Model 1		Model 2		Model 3	
	β	*t*-value	β	*t*-value	β	*t*-value
Age	−0.11	−3.39 **	−0.04	−1.42	−0.06	−1.89
Number of children	−0.03	−1.01	−0.05	−1.66	−0.05	−1.59
Never married	−0.10	−3.10	−0.10	−3.30 **	−0.10	−3.46 **
Divorced/widowed	−0.06	2.38 *	−0.08	−3.39 **	−0.08	−3.49 ***
Masculinity			0.33	14.24 ***	0.33	14.21 ***
Femininity			0.12	5.19 ***	0.12	5.19 ***
Skilled non- manual occupation					0.14	5.51 ***
Professional					0.14	5.56 ***
Adjusted *R*^2^	0.01		0.16		0.18	
*R*^2^ Change	0.01		0.15		0.02	
ANOVA (*F*-value, df)	6.58 (4,1683) ***		54.05 (6,1681) ***		46.84 (8,1679) ***	

Note: β = Standardized regression coefficient. *R*^2^ = percentage of explained variance. * *p* < 0.05; ** *p* < 0.01; *** *p* < 0.001. *t*-value = Student *t*-value
